# Adjuvant Effect of Meldrum’s Acid Derivatives on Antibiotic Activity Against Multidrug-Resistant Bacteria

**DOI:** 10.3390/ijms27198713

**Published:** 2026-09-29

**Authors:** Isaac Moura Araújo, Ewerton Yago de Sousa Rodrigues, Daniel Sampaio Alves, Clara Mariana Gonçalves Lima, Sheila Alves Gonçalves, Cícera Georgia Brito Milfont, Alisson Justino Alves da Silva, João Arthur de Oliveira Borges, Cícera Datiane de Morais Oliveira-Tintino, Samara Alves Brito, José Thyálisson da Costa Silva, Francisco Assis Bezerra da Cunha, Ricardo Andrade Rebelo, Iêda Maria Begnini, Renata Salamoni, Luiz Everson da Silva, Nathalia Monteiro Lins Freire, Renata Ferreira Santana, Thiago Mendonça de Aquino, Saulo Relison Tintino, Henrique Douglas Melo Coutinho

**Affiliations:** 1 Department of Biological Chemistry, Regional University of Cariri—URCA, Crato 63105-000, Ceará, Brazil; isaac.moura@urca.br (I.M.A.); ewerton.sousarodrigues@urca.br (E.Y.d.S.R.); daniel.sampaio10@urca.br (D.S.A.); sheila.alves@urca.br (S.A.G.); georgia.milfont@urca.br (C.G.B.M.); alisson.justino12@urca.br (A.J.A.d.S.); arthur.oliveira@urca.br (J.A.d.O.B.); datiane.morais@urca.br (C.D.d.M.O.-T.); samaralvesbrito@yahoo.com.br (S.A.B.); jose.thyalisson@urca.br (J.T.d.C.S.); cunha.urca@gmail.com (F.A.B.d.C.); saulo.tintino@urca.br (S.R.T.); 2 Department of Chemistry, Regional University of Blumenau, FURB, Blumenau 89030-903, Santa Catarina, Brazil; ricardorebelo@furb.br (R.A.R.); ieda@furb.br (I.M.B.); resalamoni13@gmail.com (R.S.); 3 Department of Chemistry, Federal University of Paraná, UFPR, Curitiba 81531-970, Paraná, Brazil; luiz_everson@yahoo.de; 4 Institute of Chemistry and Biotechnology, Federal University of Alagoas, UFAL, Maceió 57072-970, Alagoas, Brazil; nathalia.freire@icf.ufal.br (N.M.L.F.); thiago.aquino@iqb.ufal.br (T.M.d.A.); 5 Department of Nutrition, Federal University of Tocantins, Palmas 77001-090, Tocantins, Brazil; rena_nutri@yahoo.com.br

**Keywords:** bacterial resistance, Meldrum’s acid, adjuvant antimicrobial activity, membrane permeability, bacterial ATP

## Abstract

Antimicrobial resistance demands new strategies capable of restoring the effectiveness of conventional antibiotics. In this study, the antibacterial activity and adjuvant potential of five Meldrum’s acid derivatives (MAD1–MAD5) against multidrug-resistant strains of *Staphylococcus aureus* 10 and *Escherichia coli* 06 were evaluated. Minimum inhibitory concentrations (MICs) were determined by broth microdilution, and the combined effects with ampicillin, gentamicin, and norfloxacin were analyzed at subinhibitory concentrations. Membrane permeability was assessed by SYTOX Green fluorescence, and bacterial energy metabolism was assessed by quantifying intracellular ATP using bioluminescence. The derivatives did not show direct antibacterial activity (MIC ≥ 1024 µg/mL), but they significantly reduced the MICs of the antibiotics, especially gentamicin and norfloxacin. Increased membrane permeability and reduced ATP levels were most evident in *E. coli*, particularly for MAD3, MAD4, and MAD5, while *S. aureus* showed a limited response. In silico ADMET predictions indicated pharmacokinetic profiles consistent with bioactive compounds. Taken together, the results demonstrate that Meldrum’s acid derivatives act as species-dependent modulators of antimicrobial resistance, highlighting their potential as antibacterial adjuvants.

## 1. Introduction

Antimicrobial resistance is recognized by the World Health Organization as one of the greatest contemporary threats to global health, responsible for millions of difficult-to-treat infections and a growing number of annual deaths [1]. The spread of multidrug-resistant pathogens has drastically reduced the effectiveness of available antibiotics, turning once-simple infections into high-risk clinical conditions.

Among the most clinically relevant pathogens, *Staphylococcus aureus* and *Escherichia coli* present a set of virulence factors that amplify their impact on human health. *S. aureus* possesses a wide variety of toxins, such as hemolysins, leukocidins, and enterotoxins, extracellular enzymes, and adhesion proteins, which facilitate tissue invasion, immune evasion, and biofilm formation. In invasive infections, such as bacteremia, endocarditis, and necrotizing pneumonia, these factors make *S. aureus* one of the most lethal known pathogens, especially in methicillin-resistant variants (MRSA), associated with high mortality rates and serious complications [2].

In turn, *E. coli*, although part of the human gut microbiota, harbors a diversity of highly pathogenic extraintestinal pathotypes, such as uropathogenic strains (UPEC), responsible for most urinary tract infections. These pathotypes express adhesins, siderophores, toxins such as hemolysin A, and sophisticated iron acquisition mechanisms, facilitating colonization and invasion of deep tissues. The severity increases when these strains carry resistance genes, such as extended-spectrum β-lactamases (ESBLs) and carbapenemases, which not only neutralize entire classes of antibiotics but also favor rapid clonal dissemination within hospital environments [3]. Thus, the convergence of high virulence and resistance makes *E. coli* and *S. aureus* central pillars in the global antimicrobial crisis.

Given this alarming scenario, the search for new therapeutic agents that can simultaneously overcome virulence factors and resistance mechanisms becomes essential.

**Figure 1:**
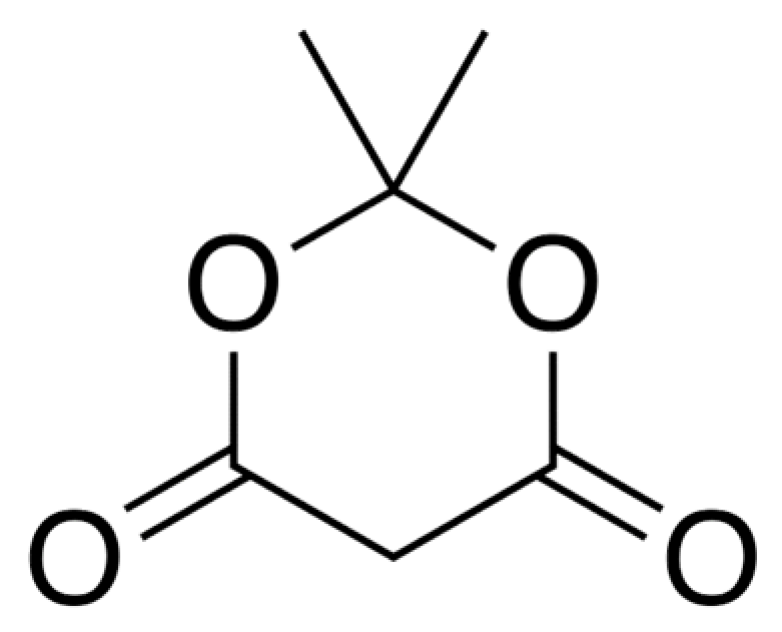
Chemical structure of Meldrum’s acid.

Meldrum’s acid (2,2-dimethyl-1,3-dioxan-4,6-dione) stands out as one of the most versatile synthetic molecules in organic chemistry due to its highly activated dioxanonic core, composed of two conjugated carbonyls and gem-dimethyl substituents (Figure 1) that confer conformational stability and pronounced acidic character (pKa ≈ 4.8–5.0) [4]. This structural combination favors the formation of enolates and reactive intermediates, such as ketenes, allowing its wide application in Knoevenagel condensations, cyclizations, and the construction of bioactive heterocyclic skeletons [5,6]. The ease of functionalizing this core has driven the generation of chemical libraries with modulable electronic and lipophilic properties, a fact that has motivated great interest in the synthesis of derivatives with pharmacological potential, including antimicrobial compounds and resistance modulators [7,8].

Several studies have demonstrated that Meldrum’s acid derivatives possess strong potential as modulators of antimicrobial resistance, acting through multiple cellular mechanisms. Certain analogs have been able to significantly enhance the activity of antibiotics, reducing the MICs of fluoroquinolones, aminoglycosides, and β-lactams against multidrug-resistant strains, including *Staphylococcus aureus* and *Escherichia coli* [9]. Studies also show that these compounds can inhibit efflux pumps, such as MepA, restoring the susceptibility of resistant isolates and altering the transport dynamics of antimicrobial compounds [10].

Molecular modeling studies and QSAR/QSRR analyses reinforce those aromatic modifications and electronegative substitutions in the Meldrum’s core modulate lipophilic properties and affinity for bacterial targets, influencing penetration and interaction with membrane components, which may explain the increased permeability observed in different strains [7,11]. Additionally, functionalized derivatives of the Meldrum’s skeleton have demonstrated the ability to suppress virulence factors and biofilm formation, expanding their potential as therapeutic adjuvants in combating bacterial resistance [12].

Thus, the study with Meldrum’s acid derivatives represents a perspective in the discovery of new antimicrobial agents and bacterial resistance modifiers. The chemical versatility of these compounds, combined with their proven activity against highly pathogenic and multidrug-resistant bacteria, reinforces their importance as candidates for innovative therapies in a global scenario where new solutions are needed.

## 2. Results and Discussion

### 2.1. Direct Activity of Meldrum’s Acid Derivatives

When analyzing the minimum inhibitory concentration of Meldrum’s acid derivatives against multidrug-resistant *S. aureus* 10 and *E. coli* 06 bacteria, MIC ≥ 1024 µg/mL results were obtained for all derivatives, which were not clinically relevant.

Studies using other Meldrum’s acid derivatives also obtained MIC ≥ 1024 µg/mL, as in the studies of Araújo et al. (2024, 2025), da Silva et al. (2021), and Sampaio et al. (2014) [9,10,13,14]; however, in the study of Bukhari et al. (2023) [7], it was observed that vanylidene derivatives of Meldrum’s acid had direct activity by broth microdilution against *Staphylococcus aureus* (ATCC 25923) [15], *Bacillus subtilis* (ATCC 6633) [16], *Bacillus cereus* (ATCC 10987) [17], and *Escherichia coli* (ATCC 25922) [18], probably due to a long alkyl chain that makes it more lipophilic and membrane permeable.

### 2.2. Adjuvant Effect of Meldrum’s Acid Derivatives on MDR Strains

**Figure 2:**
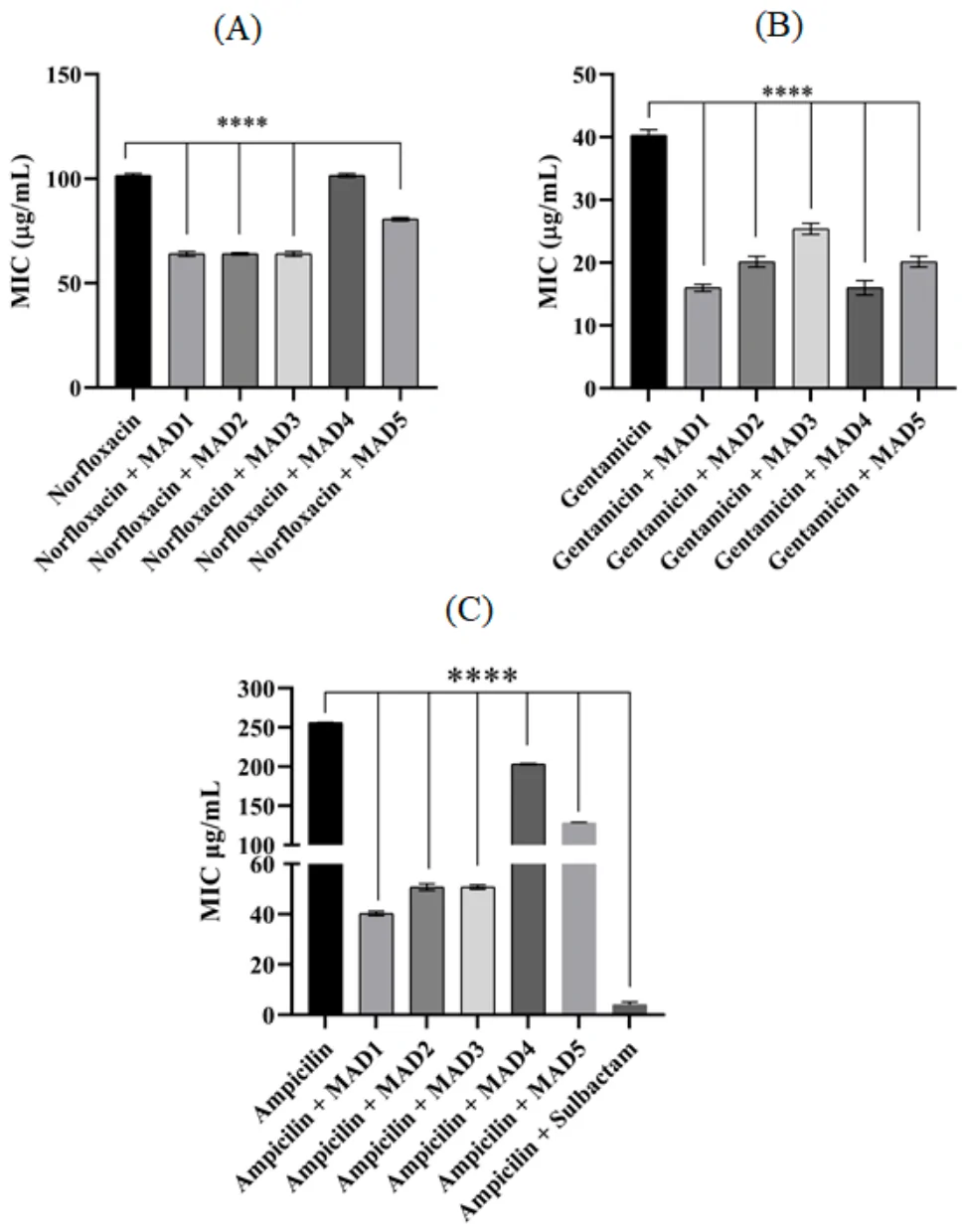
Adjuvant effect of Meldrum’s acid derivatives in combination with the antibiotics ampicillin (**A**), gentamicin (**B**) and norfloxacin (**C**) in multidrug-resistant *Staphylococcus aureus*. “****” *p* < 0.0001 indicates significant differences between groups. Statistical significance was determined by one-way ANOVA and Bonferroni posthoc test.

When administered alone, ampicillin showed a minimum inhibitory concentration (MIC) of 256 ± 0.5 µg/mL. When its effect was combined with Meldrum’s acid derivatives, the MIC required to inhibit bacterial growth decreased, in µg/mL, to 40.31 ± 0.8 with MAD1, 50.8 ± 1.3 with MAD2, 50.8 ± 0.8 with MAD3, 203.2 ± 0.8 with MAD4, and 128 ± 1.15 with MAD5. The association with sulbactam yielded an MIC of 4 ± 1.5 (Figure 2); the marked reduction in the MIC of ampicillin in the presence of sulbactam suggests that β-lactamase-mediated resistance may contribute to the reduced susceptibility of this isolate. However, this result does not directly confirm β-lactamase production or demonstrate inhibition of the enzyme by the Meldrum’s acid derivatives.

Gentamicin, when administered alone, had a MIC of 40.31 ± 0.8 µg/mL, and when combined with the derivatives, a potentiation of its effect was observed, decreasing the MIC required to inhibit bacterial growth. The combination of this antibiotic with the products yielded MICs of 16 ± 0.8, 20.15 ± 0.8, 25.4 ± 0.8, 16 ± 1.15, and 20.15 ± 0.8 µg/mL, respectively, for MAD1 to MAD5 (Figure 2).

The MIC of norfloxacin alone was 101.6 ± 0.8 µg/mL, and when combined with the derivatives, the MICs were 64 ± 1.15 with MAD1, 64 ± 0.5 with MAD2, 64 ± 1.15 with MAD3, and 80.6 ± 0.8 µg/mL for MAD5. Only the combination with MAD4 did not yield a significant result, not altering the MIC of norfloxacin, which remained at 101.6 ± 0.8 µg/mL (Figure 2).

**Figure 3:**
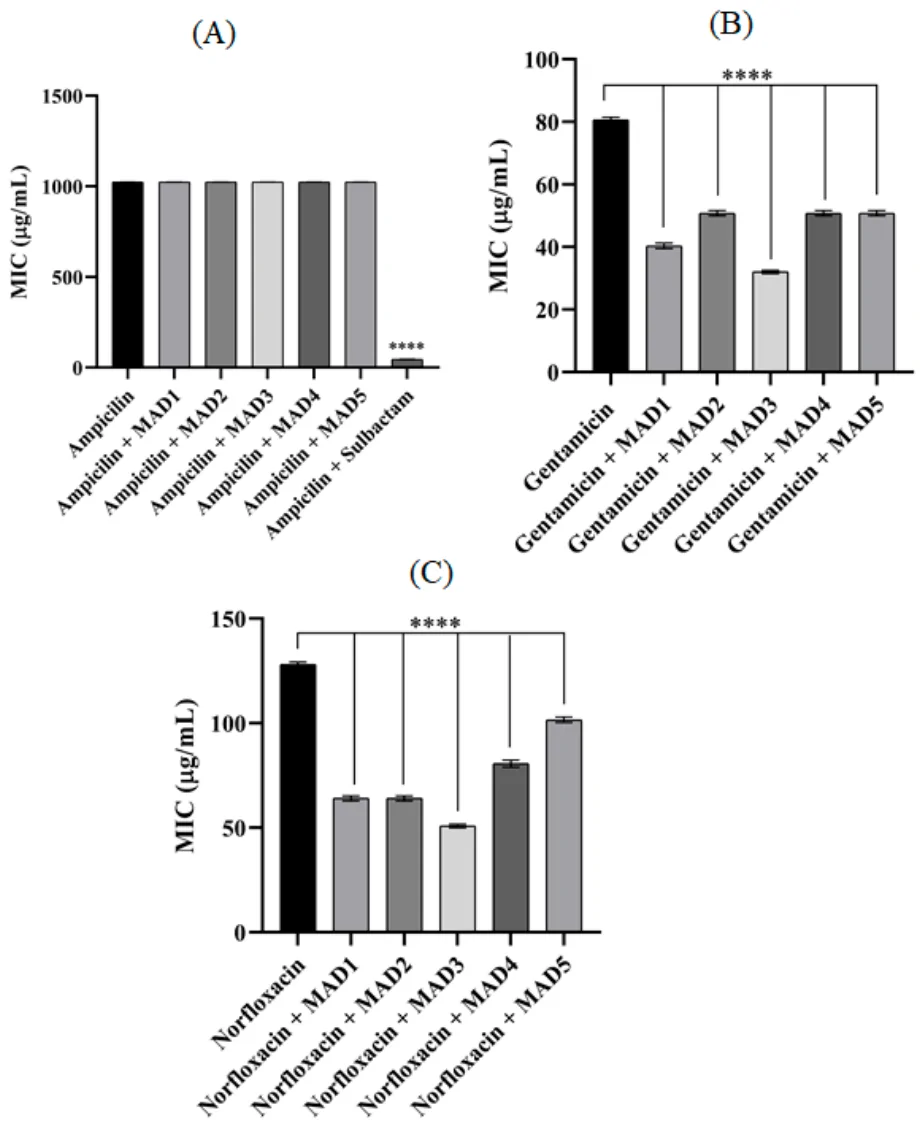
Adjuvant effect of Meldrum’s acid derivatives in combination with the antibiotics ampicillin (**A**), gentamicin (**B**) and norfloxacin (**C**) in multidrug-resistant *Escherichia coli*. “****” *p* < 0.0001 indicates significant differences between groups. Statistical significance was determined by one-way ANOVA and Bonferroni posthoc test.

The MIC of ampicillin, as well as its combination with Meldrum’s acid derivatives, was ≥ 1024 µg/mL; therefore, for this strain of *E. coli*, ampicillin did not obtain a clinically relevant result (Figure 3). The effect of penicillins, such as ampicillin, on Gram-negative bacteria like *E. coli* may be reduced due to their outer membrane and the enzymes present in the periplasmic space, such as β-lactamases [19]. The reduction observed in the presence of sulbactam is compatible with a possible contribution of β-lactamase-mediated resistance; however, direct enzymatic or molecular assays would be required to confirm this mechanism.

The association with the derivatives potentiated the effect of gentamicin; alone, it had a MIC of 80.6 ± 0.8 µg/mL, but in combination, gentamicin had its MIC reduced to 40.3 ± 0.8 (MAD1), 50.8 ± 0.8 (MAD2), 32 ± 0.5 (MAD3), 50.8 ± 0.8 (MAD4), and 50.8 ± 0.8 (MAD5) µg/mL (Figure 3).

Meldrum’s acid derivatives also potentiated the action of norfloxacin by decreasing its MIC, which alone was 128 ± 1.15 µg/mL, to 64 ± 1.15 µg/mL with MAD1, 64 ± 1.15 µg/mL with MAD2, 50 ± 0.8 µg/mL with MAD3, 80.6 ± 1.66 µg/mL with MAD4 and 101.59 ± 1.28 µg/mL with MAD5 (Figure 3).

The fold reduction in antibiotic MIC was calculated by dividing the MIC of the antibiotic alone by the MIC obtained in combination with each Meldrum’s acid derivative. The calculated values are presented in Table 1, allowing direct comparison of the magnitude of antibiotic potentiation produced by MAD1–MAD5 across the different antibiotics and bacterial strains.

This study sought to investigate the effect of 5 derivatives of Meldrum’s acid on multidrug-resistant Gram-positive and Gram-negative strains, corroborating the study, Araújo et al., (2024, 2025) [10,13], It was found that 3 derivatives of Meldrum’s acid were responsible for inhibiting the NorA and MepA efflux pumps in *S. aureus*, both by potentiating the effect of antibiotics and by increasing the fluorescence of ethidium bromide.

Another study sought to verify the association of an arylaminomethylene derivative of Meldrum’s acid with fluoroquinolones, such as norfloxacin, orfloxacin, and lomefloxacin, in multidrug-resistant strains of *S. aureus* 10, *E. coli* 06, and *P. aeruginosa* 24, and observed that this derivative potentiated all antibiotics tested in all strains [9]. Sampaio et al., 2014 [14] investigated in multidrug-resistant strains of *S. aureus* 358, *E. coli* 27 and *P. aeruginosa* 03, the effect of an aminomethylene derivative of Meldrum’s acid in combination with aminoglycosides (Amikacin, gentamicin and neomycin) obtaining satisfactory results for amikacin and gentamicin in the 3 strains tested, with neomycin, only against *S. aureus* was the MIC of the antibiotic potentiated.

The use of synthetic molecules is being widely used in an attempt to resensitize bacteria resistant to existing antibiotics. Meldrum’s acid is a compound widely used in the synthesis of heterocyclic compounds with biological activity. Silva, 2006 [20] it was found that some arylaminomethylene derivatives showed antiparasitic activity against strains of *Leishmania amazonensis* and *T. cruzi*, and it was also observed that a thiadiazole derivative showed anti-herpetic activity in strains resistant to acyclovir.

The versatility of Meldrum’s acid is also exploited in the synthesis of various heterocyclic compounds of medicinal importance, such as pyridines with anti-inflammatory activity [21], pyrimidines with antitumor activity [22] and 1,8-naphthyridines with antibiotic-potentiating and efflux pump-inhibiting activity [23,24].

Investigating different classes of antibiotics is fundamental, since each class presents distinct and complementary mechanisms of action. Penicillins inhibit peptidoglycan synthesis by binding to penicillin-binding proteins (PBPs), compromising the integrity of the bacterial cell wall. Aminoglycosides, in turn, interact with the 30S subunit of the ribosome, resulting in errors in protein translation and consequent interruption of the synthesis of essential proteins. Fluoroquinolones, on the other hand, act by inhibiting topoisomerases II and IV, enzymes responsible for modulating the structure and rejoining of DNA, culminating in genomic instability and bacterial death [25,26,27]. Furthermore, strategies that seek to reduce the concentration of antibiotics needed to achieve the therapeutic effect, such as their combined use with other bioactive compounds, can contribute to decreasing systemic toxicity and potential adverse effects associated with treatment.

Because they do not have direct antibacterial activity, it is unlikely that Meldrum’s acid derivatives act through these mechanisms; however, they can act as adjuvants by acting on other mechanisms such as cell membrane permeability, intracellular ATP production, or inhibiting efflux pumps, as described by [10,13].

### 2.3. Evaluation of Membrane Permeability Alteration by SYTOX Green Fluorescence

**Figure 4:**
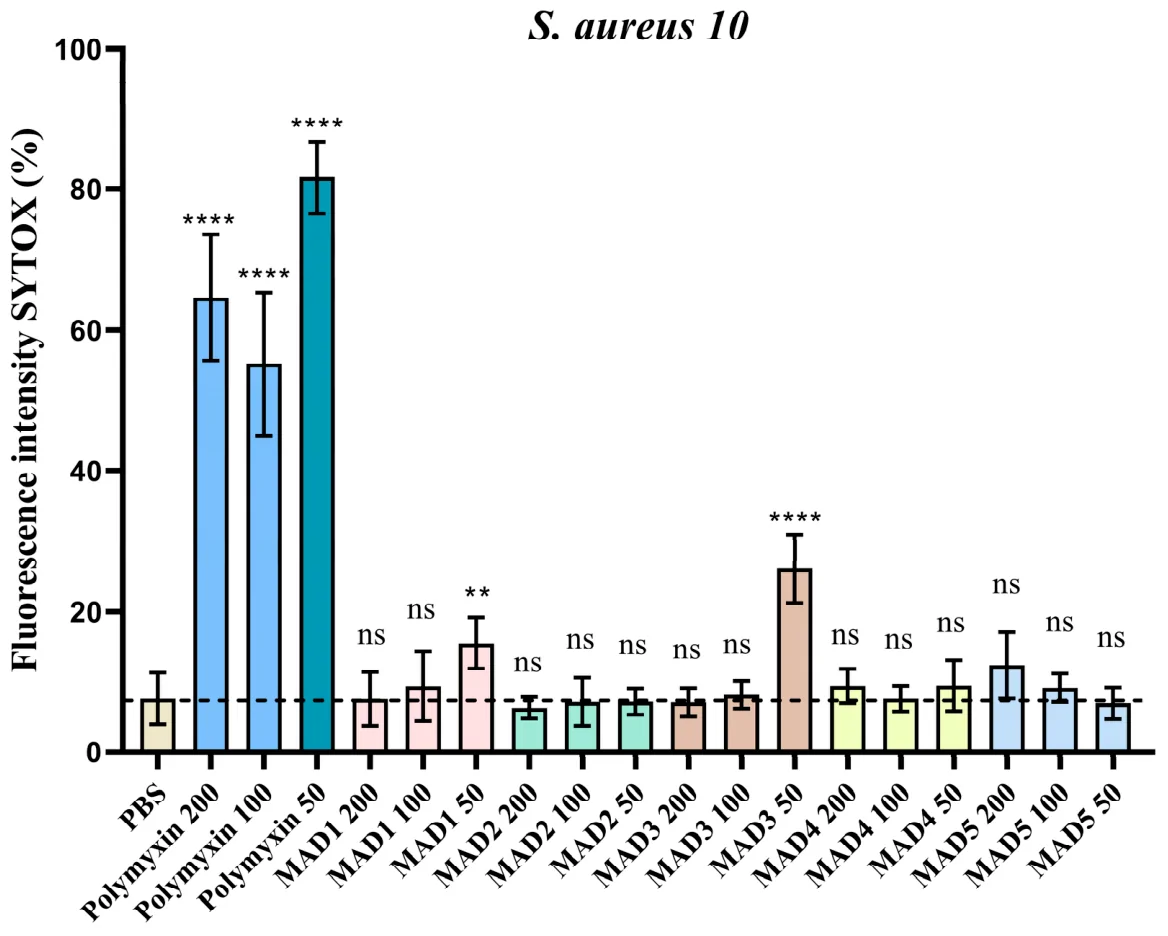
Effect of Meldrum’s acid derivatives on the fluorescence intensity of Sytox green caused by increased membrane permeability of *S. aureus* 10. “**” *p* = 0.0015, “****” *p* < 0.0001 ns: non-significant, analyzed by one-way ANOVA followed by Dunnett’s posthoc test and confirmed by non-parametric *t*-test.

The evaluation of the interaction of Meldrum’s acid derivatives with the *S. aureus* 10 membrane demonstrated that, under sub-inhibitory conditions, most of the tested concentrations did not result in a detectable increase in membrane permeability. These findings indicate that, under such conditions, the compounds do not exert a direct effect on membrane integrity, a relevant aspect for their consideration as adjuvants, since their modulating action does not appear to involve physicochemical disturbance of the cell barrier. Only the concentration of 50 µg/mL of MAD 1 and MAD3 increased membrane permeability by 7.6% and 18.45% respectively in relation to the negative control PBS (Figure 4).

The increase in polymyxin B permeability relative to PBS was 56%, 47%, and 74%, respectively, for concentrations of 200, 100, and 50 µg/mL. These findings suggest that the observed antibiotic potentiation cannot be fully explained by increased membrane permeability under the tested conditions, although a contribution of membrane-associated effects cannot be excluded, except perhaps for MADs 1 and 3.

Polymyxin B was used as a positive control because, although in clinical practice it is used against Gram-negative bacteria, under experimental conditions polymyxin can increase the permeability of Gram-positive bacteria such as *S. aureus* [28].

The study by Freitas et al., 2024 [29] showed that synthetic heterocyclic compounds such as thiadiazines increased the fluorescence of Sytox green in the *S. aureus* K2068 strain that produces the MepA efflux pump, corroborating this study, which shows that even though most concentrations did not increase fluorescence, two MADs increased it at the lowest concentration. This demonstrates that the use of synthetic heterocyclic substances has increased with the intention of reducing bacterial resistance to antibiotics.

**Figure 5:**
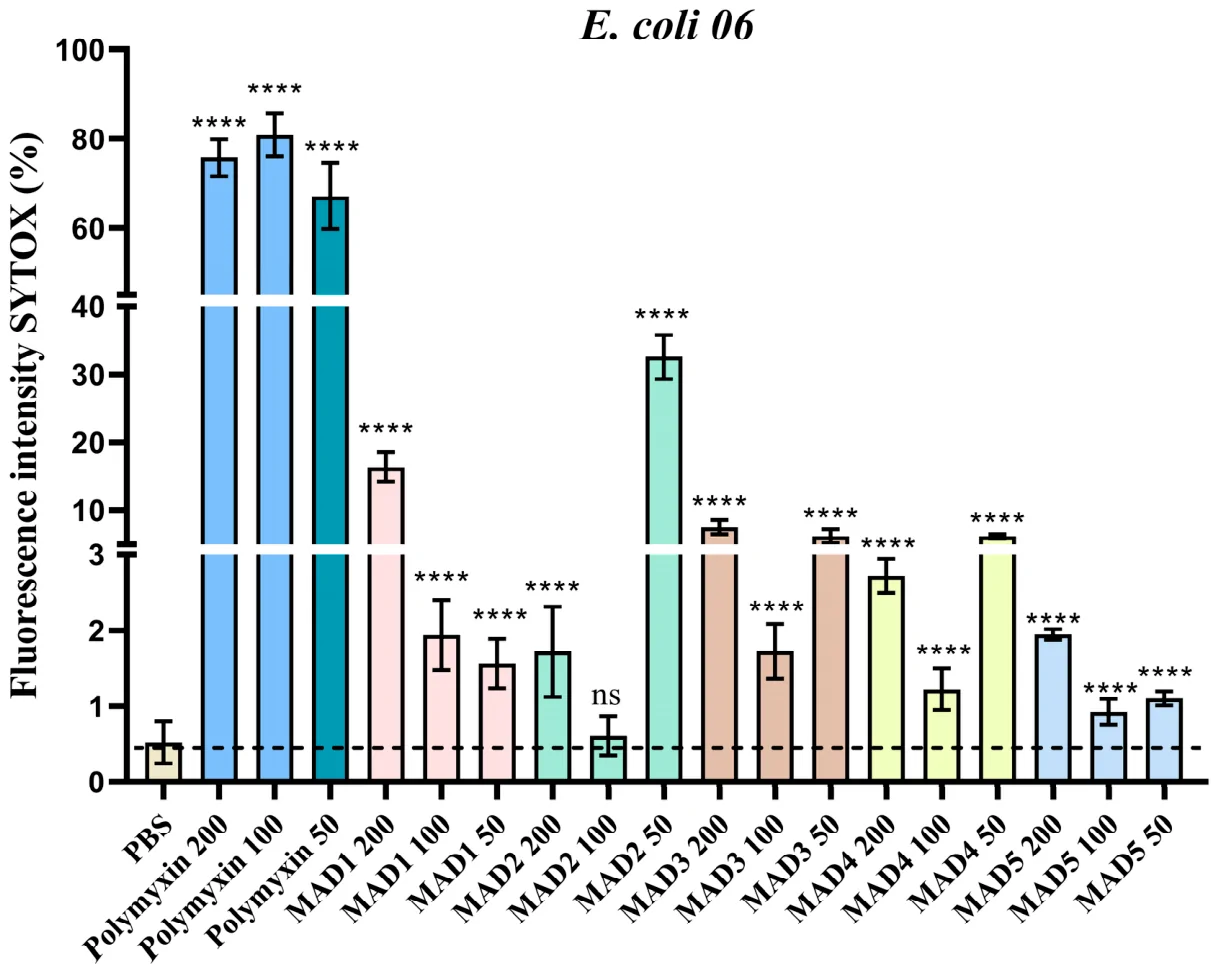
Effect of Meldrum’s acid derivatives on the fluorescence intensity of Sytox green caused by increased membrane permeability of *E. coli* 06. “****” *p* < 0.0001 ns: non-significant, analyzed by one-way ANOVA followed by Dunnett’s posthoc test and confirmed by non-parametric *t*-test.

When evaluating the alteration of Sytox green fluorescence by Meldrum’s acid derivatives, it was observed that all derivatives increased the intensity of Sytox green fluorescence, consistent with increase in the permeability of the *E. coli* membrane (Table 2, Figure 5). Only the concentration of 100 µg/mL of MAD2 did not increase the intensity compared to PBS, whose fluorescence was 0.51%. The most significant increases were observed at concentrations of 200 µg/mL of MAD1 and 50 µg/mL of MAD3, which increased fluorescence by 15.83% and 32.04%, respectively.

A study verified that the use of two heterocyclic compounds (13 and 1771) based on 1,3,4-oxadiazole increased the fluorescence of 4 g-negative bacterial strains, *E. coli* K2, *P. aeruginosa* PAO1, *K. pneumoniae* 699 and *A. baumannii* DF1000, when combined with PAβN, a well-studied efflux pump inhibitor that also permeabilizes the outer membrane of Gram-negative bacteria [30].

In Gram-negative bacteria, polymyxins, such as colistin, exert bactericidal activity upon reaching LPS in the cytoplasmic membrane. Binding to LPS results in the displacement of cationic bridges and the rupture of the cytoplasmic membrane, which ultimately leads to permeability, culminating in the loss of cytoplasmic contents, cell lysis, and bacterial death [31].

This study is necessary because studies involving Meldrum’s acid derivatives are scarce, especially those involving some type of mechanism of action such as membrane permeabilization.

### 2.4. Assay of the Interaction with Intracellular ATP by Luciferase Bioluminescence

The quantification of intracellular ATP levels was performed using a bioluminescence system based on the ATP-luciferin-luciferase reaction, after exposing *Staphylococcus aureus* and *Escherichia coli* bacteria to Meldrum’s acid derivatives at subinhibitory concentrations (MAD1–MAD5, 200 µg/mL). The values presented were corrected by subtracting the blank and expressed in relative luminescence units (RLU). The group designated “ATP” corresponded to the positive technical control, containing exogenous ATP added to the reaction, used to confirm luciferase activity. CCCP was used as a positive control for membrane potential collapse and ATP depletion [32], while the negative control consisted of untreated bacterial cultures.

For *S. aureus*, the negative control showed an average signal of 33 RLU, and the ATP control reached 986 RLU, confirming the efficiency of the enzymatic system. The CCCP resulted in 17 RLU, indicating no significant reduction in ATP content compared to the negative control. Samples treated with MAD1–MAD5 derivatives exhibited the following average values: 28, 94.5, 26, 20, and 39 RLU, respectively. Although MAD2 showed a slight increase in signal, statistical analysis did not reveal significant differences between the compounds and the negative control. These results suggest that, under the tested conditions, Meldrum’s acid derivatives did not substantially interfere with the bioenergetics of *S. aureus*. This behavior may be related to the absence of increased membrane permeability, since maintaining membrane integrity is essential to preserve the electrochemical proton gradient and, consequently, energy transduction processes such as ATP generation and active transport [33].

This behavior can be attributed to the typical structural barrier of Gram-positive bacteria, whose thick peptidoglycan and lower membrane fluidity hinder the diffusion of lipophilic or partially ionized compounds, reducing the penetration of uncoupling agents such as CCCP [34,35]. Furthermore, the organization of the electron transport system and the lower dependence on proton-motive force in some metabolic pathways of *S. aureus* may also contribute to a lower sensitivity to compounds that alter the proton gradient [36].

In contrast, for *E. coli*, a more heterogeneous response profile was observed. The negative control showed 110 RLU, while the ATP group reached 213 RLU, confirming the integrity of the luciferase reaction. CCCP significantly reduced luminescence to 36 RLU, consistent with its expected effect on the proton-motive force and intracellular ATP levels. Among the derivatives tested, MAD1 (178 RLU) and MAD2 (164 RLU) did not differ statistically from the negative control, suggesting an absence of significant metabolic impact. However, MAD3 (52 RLU), MAD4 (34 RLU), and MAD5 (74 RLU) exhibited considerably lower values, the reduced luminescence observed for MAD3, MAD4, and MAD5 was close to that obtained with CCCP, suggesting that these derivatives may affect bacterial energy homeostasis. However, the present assay does not allow discrimination between altered ATP levels, membrane-associated effects, or other metabolic changes.

These findings are consistent with the fact that *E. coli*, a Gram-negative bacterium, has an outer membrane rich in phospholipids and porins that facilitate the diffusion of amphiphilic compounds, making it more susceptible to the action of agents that disrupt the integrity of the bilayer [37]. Previous studies demonstrate that CCCP acts directly by dissipating the transmembrane electrochemical gradient and inhibiting oxidative phosphorylation, leading to a rapid drop in ATP levels [38,39,40].

**Figure 6:**
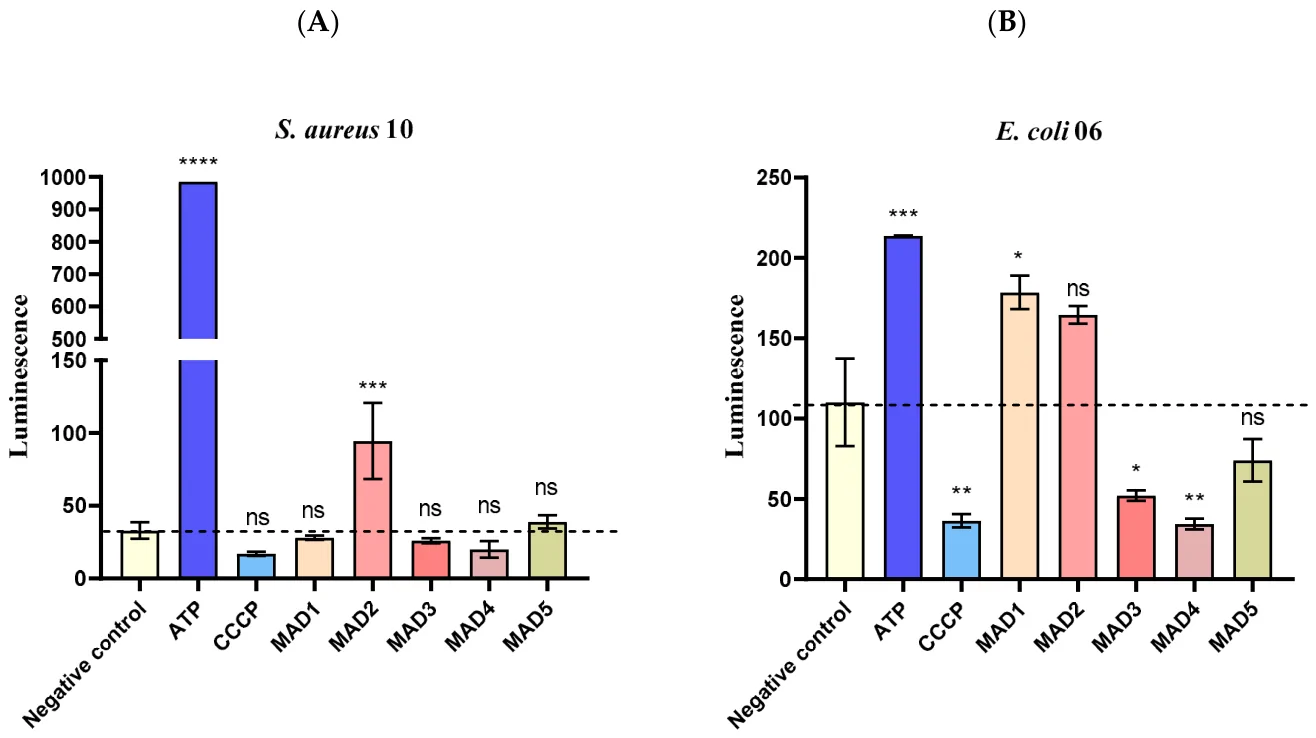
Intracellular ATP levels measured by bioluminescence (RLU) in *Staphylococcus aureus* (**A**) and *Escherichia coli* (**B**) after exposure to Meldrum’s acid derivatives (200 µg/mL, 1 h). “****” *p* < 0.0001, “***” *p* = 0.0002, “**” *p* = 0.003, “*” *p* = 0.01, ns = non-significant, analyzed by one-way ANOVA followed by Dunnett’s posthoc test and confirmed by non-parametric *t*-test.

Therefore, the results obtained indicate that Meldrum’s acid derivatives (MAD1–MAD5) exhibit distinct behaviors towards the two bacterial species. While *S. aureus* showed a comparatively limited change in ATP-associated luminescence, *E. coli* showed sensitivity to some derivatives, particularly MAD3 and MAD4, suggesting that the observed changes in ATP-associated luminescence may differ according to bacterial cell-envelope characteristics and compound susceptibility., since it is in the bacterial membrane that the ATP production proteins are found [41]. These structural differences between Gram-positive and Gram-negative bacteria, coupled with the lipophilic nature of the derivatives, support the hypothesis that the effects of the derivatives may differ between bacterial species, potentially reflecting differences in compound uptake, membrane properties, and cellular responses. (Table 3, Figure 6).

### 2.5. ADMET Predictions

The pharmacokinetic properties predicted by SwissADME [42] for the five molecules reveal a general profile compatible with compounds of bioactive potential (Table 4). Overall, most molecules presented physicochemical parameters within the limits accepted by the classical rules of Lipinski, Veber, Egan, Ghose, and Muegge, suggesting conformity with drug-likeness criteria and indicating a good probability of being orally active; however, MAD2 and MAD5 showed one violation of the Ghose criteria (Table 5). This behavior is relevant, since the maintenance of a moderate number of hydrogen bond acceptors and donors, associated with a molecular mass of less than 500 Da and a moderate surface polarity, favors both gastrointestinal absorption and systemic bioavailability of the compounds [43].

**Figure 7:**
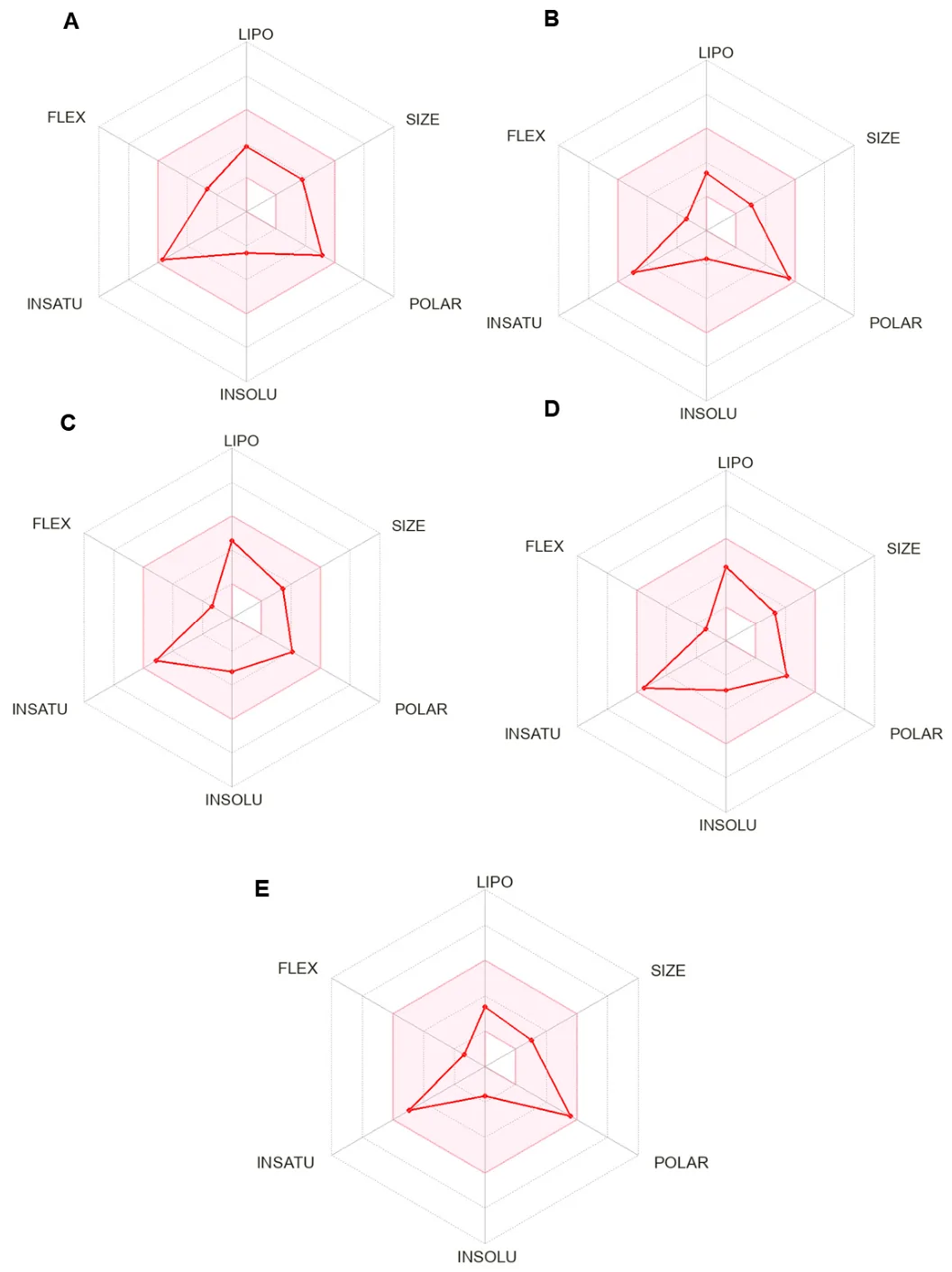
Radar of physicochemical properties predicted by SwissADME for MADs: (**A**) MAD1, (**B**) MAD2, (**C**) MAD3, (**D**) MAD4 and (**E**) MAD5. Each radar represents the distribution of the descriptors LIPO, SIZE, POLAR, INSOLU, INSATU and FLEX, highlighting structural differences and variations in the drug-likeness profile between the molecules.

MAD1 exhibited a noticeable increase in surface polarity and a slight reduction in lipophilicity compared to MAD3, implying an even more water-soluble profile. This characteristic is associated with potentially lower intestinal absorption, although sufficient to allow oral bioavailability at therapeutic levels [44]. In the radar of MAD1 molecule properties (Figure 7A), an overall profile still compatible with the drug space is observed, but with some important nuances in relation to MAD3. The LIPO region shows a slight reduction, indicating a slightly lower lipophilicity; together with the slight increase in SIZE and POLAR, this suggests a marginally more water-soluble and slightly more voluminous molecule, which may favor dissolution in aqueous medium, albeit at the cost of slightly lower passive permeability. The INSOLU axis remains in an intermediate range, indicating that, despite the gain in polarity, MAD1 does not become excessively hydrophilic, preserving a balance between solubility and permeability [45].

Another relevant aspect is the behavior along the INSATU and FLEX axes. The INSATU contribution is more pronounced, reflecting a higher proportion of unsaturations and, consequently, an increase in structural rigidity, while FLEX remains at a moderate level, with the number of rotations still controlled. This combination of high unsaturation and contained flexibility suggests a structurally more rigid molecule than MAD3, which may reduce the entropic cost of binding and favor more defined interactions with the biological target. Taken together, the radar indicates that MAD1 occupies a region of the chemical space that is slightly more polar and less lipophilic than MAD3, but still within a profile considered predicted to display physicochemical features compatible with oral drug-like space [46].

MAD2 exhibits a physicochemical profile strongly influenced by high polarity and low lipophilicity, as evidenced by the reduced logP values and the large polar surface area. This set of characteristics gives the molecule a markedly hydrophilic nature, also reflected in the predictions of high aqueous solubility. This behavior favors dissolution in biological media and supports the prediction of high gastrointestinal absorption, although the pronounced polarity tends to limit passive diffusion through lipid membranes. Consistently, MAD2 was predicted by SwissADME to have low probability of BBB permeation, indicating a pharmacokinetic profile predominantly directed towards peripheral circulation.

The property radar analysis reinforces this interpretation by revealing a pronounced expansion in the POLAR axis, accompanied by significant contraction in the LIPO and INSOLU axes, evidencing the predominance of hydrophilic characteristics in the molecular architecture of MAD2 (Figure 7B). The SIZE axis remains within the range considered adequate for oral drug candidates, suggesting that increased polarity does not translate into a relevant molecular volume penalty or steric impact. Additionally, the moderate contribution to the INSATU axis indicates the presence of unsaturated systems that confer a certain degree of structural rigidity, while the low value observed in FLEX reflects a reduced number of rotational linkages. This combination of conformational rigidity and controlled flexibility may contribute to more defined conformations and lower entropic cost of binding to the biological target. From a metabolic point of view, MAD2 presents a particularly favorable profile, without relevant warnings for inhibition of the main cytochrome P450 isoforms or P-gp-mediated efflux, reinforcing its potential for peripheral therapeutic applications.

MAD3 exhibited high solubility characteristics and a low logP value, indicating a relatively hydrophilic nature. This behavior tends to facilitate dissolution in aqueous medium, an essential factor for intestinal absorption, but it can reduce passive permeability through biological membranes. The moderate polar surface area (TPSA) reinforces this duality, as it remains within the range considered ideal for absorption, but slightly above the limit suggested for penetration into the central nervous system. Thus, it is observed that MAD3 tends to be absorbed orally, but restricted to peripheral circulation, and was predicted by SwissADME to have low probability of BBB permeation based on its physicochemical profile, since higher TPSA values correlate with the absence of cerebral permeability.

The radar profile generated by SwissADME for the MAD3 molecule (Figure 7C) shows an adequate balance between the main properties determining oral activity, distributed along the axes of lipophilicity (LIPO), molecular size (SIZE), polarity (POLAR), solubility (INSOLU), unsaturation (INSATU), and flexibility (FLEX). Overall, the molecule shows a consistent drug space, indicating a favorable combination of physicochemical characteristics for the development of bioactive candidates. The LIPO projection reveals moderate lipophilicity, consistent with compounds that are well absorbed in the intestine, while the solubility region (INSOLU) demonstrates satisfactorily soluble behavior, suggesting potential for adequate dissolution in biological media. The POLAR axis dimension remains within ideal limits, reflecting a balance between hydrogen bonding capacity and permeability to biological membranes [47]. Furthermore, the low degree of structural flexibility (FLEX) indicates molecular rigidity compatible with good conformational stability, a characteristic that favors selective recognition at target sites. In general, the property radar analysis suggests that MAD3 has a harmonious physicochemical profile compatible with drug-likeness parameters, suggesting physicochemical characteristics compatible with commonly used drug-likeness criteria.

In contrast, MAD4 showed the greatest balance between lipophilicity and solubility among the compounds evaluated, configuring a more harmonious profile within the pharmacophoric range recommended for oral candidates. This relationship indicates a high intestinal absorption potential and moderate permeability, while preserving good compatibility with the oral route. The absence of alert for P-glycoprotein (P-gp) substrate further suggests that the compound may escape cellular efflux mechanisms, increasing its systemic availability (Table 4).

All evaluated derivatives presented a PAINS alert associated with the ene_six_het_A structural pattern (Table 5). This finding indicates a potential risk of assay interference and should therefore be considered when interpreting the biological results. However, the presence of a PAINS alert does not by itself demonstrate that the observed biological activity is nonspecific or artifactual. Experimental validation using orthogonal assays will be necessary to further assess the specificity of the observed effects.

The graphical analysis of the radar surface reinforces these findings, showing a more uniform geometric outline compared to other molecules. On the LIPO axis, the projection reaches intermediate values, indicating sufficient lipophilicity to cross biological membranes, but without exceeding the limit associated with low solubility—a result that aligns with its position on the INSOLU axis, where MAD4 shows moderate displacement, reflecting adequate solubility [43]. The POLAR axis reveals controlled polarity, indicating a balanced presence of functional groups capable of hydrophilic interactions without compromising passive transport (Figure 7D).

Another relevant point is the positioning of MAD4 on the INSATU and FLEX axes. The elongated surface in INSATU suggests the presence of a relatively unsaturated core, conferring structural rigidity that reduces the number of accessible conformations and tends to favor more well-defined interactions with biological targets [48]. Simultaneously, the subtle indentation in FLEX indicates a limited number of rotational bonds, avoiding excessive conformational flexibility—a characteristic associated, in previous studies, with a reduction in entropic penalties during molecular coupling. Thus, the balanced and symmetrical shape of the radar surface demonstrates that MAD4 occupies a privileged position within the physicochemical drug space, supporting its potential as one of the most compatible compounds for oral development within the analyzed series.

Finally, MAD5 exhibits a physicochemical profile strongly inclined towards hydrophilicity. Radar surface analysis reveals a conformation more displaced towards the POLAR axis (Figure 7E), highlighting the predominance of hydrophilic characteristics in its molecular architecture. This expansion in the polarity axis is accompanied by retraction in the LIPO and INSOLU regions, clearly demonstrating the less lipophilic and more soluble character of the compound. The SIZE axis remains within an appropriate range, suggesting that, despite the high polarity, the molecule does not suffer a significant penalty in terms of molecular volume or steric impact.

The INSATU and FLEX axes show a particularly relevant behavior: the more pronounced projection in INSATU indicates the presence of unsaturated systems that confer structural rigidity to the molecule, while the intermediate level of FLEX suggests a moderate number of rotational bonds. This combination of controlled rigidity and flexibility tends to favor more defined conformations, reducing the entropic cost of binding and potentially contributing to more stable interactions with biological targets. Thus, the radar geometry of MAD5 highlights a molecule with physicochemical properties strongly inclined towards hydrophilicity, yet still capable of maintaining solubility and conformation favorable to pharmaceutical development. Although its high polarity limits its passive diffusion and systemic permeability, its simultaneously stable metabolic profile and compatibility with intestinal absorption suggest that MAD5 may be more suitable for peripheral therapeutic applications, maintaining a structural balance consistent with its eminently polar nature [49].

In general, the five molecules exhibit drug-likeness consistent with the desirable standards for drug candidate compounds, maintaining acceptable values of molecular mass, number of rotatable bonds, and surface polarity. The predicted bioavailability scores were similar among the compounds, ranging from 0.55 to 0.56. and average synthetic accessibility (~3) reinforce the chemical and pharmacokinetic viability of the structures. However, the presence of functionally reactive groups, associated with the possible inhibition of cytochrome P450 enzymes, justifies the need for complementary experimental evaluations to confirm the stability and safety of the predicted pharmacokinetic profile.

In addition, the Meldrum’s derivatives (MADs) exhibited similar predicted toxicological profiles (Table 6), although specific differences were identified and may impact their viability as antibacterial agents. Regarding target organ toxicity, hepatotoxicity was indicated as active only for compounds MAD2 and MAD5, while the others were classified as inactive. This distinction is considered relevant, the absence of a predicted hepatotoxicity signal for MAD1, MAD3, and MAD4 may represent a favorable computational feature; however, this prediction alone is insufficient to infer a therapeutic margin or safety profile [50]. Additionally, nephrotoxicity and respiratory toxicity were predicted to be active for all MADs evaluated, suggesting the presence of a possible adverse effect common to the class, which demands attention in subsequent phases of structural optimization and safety evaluation [51].

The toxicological predictions generated by ProTox 3.0 should be interpreted as preliminary computational estimates rather than experimental evidence of toxicity. The predicted nephrotoxicity and respiratory toxicity for all derivatives, as well as the predicted clinical toxicity, indicate potential safety concerns that warrant further investigation. Hepatotoxicity was predicted for MAD2 and MAD5, whereas the remaining derivatives were classified as inactive for this endpoint. These predictions do not establish the occurrence or severity of toxicity in biological systems and should therefore be interpreted cautiously. Experimental cytotoxicity and toxicological studies will be required to determine the actual safety profiles of these compounds.

The assessment of the toxicity points of Meldrum’s derivatives (MADs) showed a predominance of predictions classified as inactive for most of the molecular targets analyzed, suggesting a low propensity of these compounds for nonspecific interaction with receptors. This profile may be associated with a more targeted antibacterial mechanism of action. However, parameters related to blood–brain barrier (BBB) permeability, as well as clinical and nutritional toxicity, indicate that MADs should be considered with caution regarding potential therapeutic applications.

With regard to nuclear signaling pathways, essential regulatory pathways involved in the cellular stress response, and molecules initiating adverse events, it was observed that, predominantly, MADs did not show the predicted capacity to interact with these systems. This finding reinforces the hypothesis that these acids may act through more specialized mechanisms, such as interaction with specific target proteins, such as gyrase B and topoisomerase II [7]. Concomitantly, derivatives that exhibit lower predictive risk of hepatotoxicity and broad inactivity against these critical toxicological pathways tend to stand out as more promising candidates for future investigations and subsequent development stages.

Overall, the SwissADME and ProTox 3.0 analyses provide preliminary computational insights into the physicochemical, pharmacokinetic, and toxicological profiles of MAD1–MAD5. These predictions may assist in prioritizing compounds for further investigation but should not be interpreted as experimental evidence of efficacy, bioavailability, or safety.

## 3. Materials and Methods

### 3.1. Microorganisms

The bacterial isolates used in this study were *Staphylococcus aureus* 10 and *Escherichia coli* 06, obtained from the microorganism collection of the Laboratory of Microbiology and Molecular Biology (LMBM) of the Regional University of Cariri (URCA). The origin and antimicrobial resistance profiles of these isolates have been previously described by Bezerra et al. [52]. Both isolates were previously characterized as multidrug-resistant based on their reported antimicrobial susceptibility profiles. Before the experiments, the isolates were cultured on brain heart infusion (BHI) agar and maintained in a bacteriological incubator at 37 °C for 24 h.

### 3.2. Substances

The antibiotics Ampicillin, gentamicin, norfloxacin, sulbactam and resazurin were obtained from Sigma Aldrich Inc., St. Louis, MO, USA. Meldrum’s acid derivatives were synthesized and provided by Professor Dr. Ricardo Andrade Rebelo from the Federal University of Blumenau—FURB (Table 7) [53,54].

The antibiotics and Meldrum’s acid derivatives were dissolved in 5% dimethyl sulfoxide (DMSO) and subsequently in sterile water until a final concentration of 1024 μg/mL was obtained.

### 3.3. Determination of the Minimum Inhibitory Concentration (MIC)

The Minimum Inhibitory Concentration (MIC) of the Meldrum’s acid derivatives was determined by a serial microdilution assay [55]. 100 μL of bacterial inoculum suspended in saline solution corresponding to 0.5 on the McFarland scale was used, followed by the addition of 900 μL of brain heart infusion (BHI) culture medium in an Eppendorf tube to obtain a volume of 1 mL. A sterile 96-well microdilution plate was used to perform the assay. The plate was numerically filled by adding 100 μL of the distribution solution to each well, and then serial dilutions were made with 100 μL of the test solution, with final concentrations ranging from 512 to 8 μg/mL, up to the penultimate well, as the last well was reserved for controlling microbial growth. The plates were then incubated for 24 h at 35 °C.

For the plate readings, 20 μL of resazurin were added, and after 1 h at room temperature, a color change in the culture medium from blue to pink was observed, indicating the presence of bacterial growth, while remaining blue indicated the absence of growth. All experiments were performed in triplicate.

### 3.4. Analysis of Antibacterial Activity in Association with Antibiotics

For the analysis of combined activity, Meldrum’s acid derivatives were used at subinhibitory concentrations (MIC/8). The control group consisted of the bacterial inoculum, diluted in a final volume of 150 µL of physiological saline and added to a microtube containing 1.350 mL of BHI. In the evaluation of the substances, a solution was prepared containing 150 µL of each inoculum, the Meldrum’s acid derivatives at MIC/8 (188 µL), and the final volume supplemented with BHI (1162 mL). A 100 µL aliquot from each tube was distributed into 96-well microplates and microdiluted with 100 µL of antibiotic (ampicillin, gentamicin, or norfloxacin) at an initial concentration of 1024 µg/mL. Serial dilutions were then performed to obtain solutions with concentrations ranging from 512 to 0.5 µg/mL. Ampicillin + sulbactam was tested for the presence of the β-lactamase enzyme. The plates were incubated at 37 °C for 24 h. After incubation, resazurin was added and used as an indicator of bacterial growth. The MIC was defined as the lowest concentration capable of inhibiting bacterial growth [56]. All experiments were performed in triplicate.

### 3.5. Evaluation of Bacterial Membrane Permeability by the Fluorescence Method with SYTOX Green

For this test, the dye SYTOX Green, a DNA intercalating agent, was used. Bacterial inocula of the strains *S. aureus* 10 and *E. coli* 06 were prepared at the 1.5 McFarland concentration. 50 µL of inoculum was distributed in a 96-well black plate. Also, 50 µL of the 5 derivatives of Meldrum’s acid were added at final concentrations of 200, 100, and 50 µg/mL. Polymyxin B was used as a positive control at concentrations of 200, 100, and 50 µg/mL. Phosphate-Buffered Saline (PBS) was added to the negative control group. The plates were incubated for 1 h. Then, 100 µL of SYTOX Green was added at a final concentration of 1 µM. The plates were incubated for 1 h and fluorescence readings were performed using a Cytation 1 plate reader, BioTek^®^ (Winooski, VT, USA) and Gen5™ 3.11 software. An excitation filter of 485 nm and an emission filter of 528 nm were used. The percentage change in fluorescence was calculated relative to the PBS negative control according to the following equation: % fluorescence change = [(Fsample − FPBS)/FPBS] × 100, where Fsample represents the fluorescence intensity of each treatment and FPBS represents the fluorescence intensity of the PBS control. The tests were performed in triplicate [57].

### 3.6. Assay of the Interaction with Intracellular ATP by Luciferase Bioluminescence

The determination of intracellular ATP levels was performed by the bioluminescence method, using the commercial kit Adenosine 5′-Triphosphate (ATP) Determination Kit (A22066) from Invitrogen (Carlsbad, CA, USA), with adaptations. The bacterial strains *Staphylococcus aureus* 10 *and Escherichia coli* 06 were cultured and standardized to a turbidity equivalent to 6.0 on the McFarland scale. The inocula were then exposed to Meldrum’s acid derivatives at a concentration of 200 µg/mL and incubated for 1 h at 37 °C. After treatment, the samples were centrifuged at 10,000 rpm for 5 min at 4 °C, washed with ice-cold PBS, and centrifuged again under the same conditions to completely remove the supernatant. The cells were then resuspended in 200 µL of lysozyme solution and incubated for 20 min for enzymatic lysis. Lysozyme inactivation was performed by heating the samples to 100 °C, followed by centrifugation at 12,000 rpm for 5 min at 4 °C. 100 µL aliquots of the supernatant were transferred to a 96-well plate, the luminescence was read using a Cytation 1 microplate reader (BioTek, Winooski, VT, USA) with Gen5™ 3.11 software to remove the “blank,” and subsequently 100 µL of luciferin-luciferase solution prepared according to the manufacturer’s instructions was added and read again. All steps were performed under low light to avoid signal degradation. The emitted light intensity (RLU) was proportional to the residual cellular ATP content. CCCP was used at a final concentration of 200 µg/mL, corresponding to the concentration used for the Meldrum’s acid derivatives. Lysozyme was prepared by dissolving 2.5 mg in 3 mL. Luminescence values were corrected by subtracting the blank signal. No further normalization to bacterial biomass, optical density, viable cell counts, or total protein was performed.

### 3.7. In Silico ADMET Analysis

The two-dimensional structures of the compounds were initially drawn in ChemDraw Ultra software (version 10). Then, each structure was converted to its respective SMILES code (Table 8) and imported into the SwissADME platform. The prediction of physicochemical properties, medicinal chemistry parameters, and ADME profile was performed using SwissADME, a freely accessible tool available at: http://www.swissadme.ch accessed on 8 June 2026.

Toxicological aspects were evaluated using the free ProTox 3.0 platform (https://tox.charite.de/protox3/ accessed on 8 June 2026), a tool that allows for the prediction of a comprehensive toxicological profile based on multiple criteria. This analysis includes, among other parameters, acute toxicity, potential toxic effects on specific organs, clinical manifestations associated with toxicity, initiating events at the molecular level, and the identification of pathways related to adverse outcomes [58].

### 3.8. Statistical Analysis

The results of the experiments were expressed as geometric mean ± standard deviation, statistically evaluated by analysis of variance (One-way ANOVA) followed by the Bonferroni post-test using GraphPad Prism 8.4.3 software. Differences were considered significant when *p* < 0.05.

## 4. Conclusions

Meldrum’s acid derivatives did not exhibit direct antibacterial activity but acted as effective modulators of antibiotic activity against multidrug-resistant strains of *Staphylococcus aureus* and *Escherichia coli*. The reduction in MICs, especially for gentamicin and norfloxacin, highlights their potential as therapeutic adjuvants. The effects were species-dependent, being more pronounced in *E. coli*, where increased membrane permeability and a significant reduction in intracellular ATP were observed, suggesting interference in cellular bioenergetics. In silico analyses provided preliminary information regarding the physicochemical, pharmacokinetic, and toxicological profiles of the derivatives, highlighting features that warrant further experimental investigation. These findings contribute to the understanding of the mechanisms involved in the modulation of bacterial resistance and support the potential of Meldrum’s acid derivatives as promising tools in combinatorial therapeutic strategies.

## Figures and Tables

**Table 1:** Fold reduction in antibiotic MICs in the presence of Meldrum’s acid derivatives.

Bacteria	Antibiotic	MIC Alone (µg/mL)	MAD1	MAD2	MAD3	MAD4	MAD5
*S. aureus* 10	Ampicillin	256	6.35×	5.04×	5.04×	1.26×	2.00×
*S. aureus* 10	Gentamicin	40.31	2.52×	2.00×	1.59×	2.52×	2.00×
*S. aureus* 10	Norfloxacin	101.6	1.59×	1.59×	1.59×	1.00×	1.26×
*E. coli* 06	Gentamicin	80.6	2.00×	1.59×	2.52×	1.59×	1.59×
*E. coli* 06	Norfloxacin	128	2.00×	2.00×	2.56×	1.59×	1.26×

Note: Fold reduction was calculated as MIC of the antibiotic alone divided by the MIC of the antibiotic in combination with each Meldrum’s acid derivative.

**Table 2:** Percentage difference in fluorescence of polymyxin Sytox green and Meldrum’s acid derivatives compared to PBS.

%	Polymyxin-B	MAD1	MAD2	MAD3	MAD4	MAD5
200 µg/mL	75.20	15.83	1.197	6.983	2.198	1.428
100 µg/mL	80.28	1.419	NS	1.204	0.7062	0.4040
50 µg/mL	66.62	1.041	32.04	5.656	5.658	0.5862

**Table 3:** Effect of Meldrum’s acid derivatives on intracellular ATP levels of multidrug-resistant *Staphylococcus aureus* and *Escherichia coli*, expressed as relative luminescence units (RLU).

Group/Condition	*S. aureus* 10 (RLU)	*E. coli* 06 (RLU)
Negative control (no treatment)	33	110
Technical control (ATP + luciferase)	986	213
CCCP (positive control)	17	36
MAD1	28	178
MAD2	94.5	164
MAD3	26	52
MAD4	20	34
MAD5	39	74

**Table 4:** Physicochemical properties of MAD1–5 molecules, calculated by SwissADME. Predictions include aspects related to absorption, distribution, metabolism, and excretion (ADME), highlighting differences in polarity, lipophilicity, and bioavailability potential among the compounds.

Molecule	Molecular Formula	n. HA	F. Csp3	TPSA (Å^2^)	Log P_o/w_	GI Absorption	BBB Permeant	P-gp Substrate
MAD1	C_14_H_15_N_3_O_5_	22	0.29	106.62 Å^2^	1.08	High	No	No
MAD2	C_9_H_11_N_5_O_4_	18	0.44	108.23 Å^2^	0.15	High	No	No
MAD3	C_14_H_16_N_2_O_4_	20	0.36	77.52 Å^2^	2.00	High	No	No
MAD4	C_13_H_14_N_2_O_4_	19	0.31	77.52 Å^2^	1.66	High	No	No
MAD5	C_8_H_9_N_5_O_4_	17	0.38	119.09 Å^2^	0.03	High	No	No

n. HA: Number heavy atoms.

**Table 5:** Pharmacological properties and medicinal chemistry of MAD1–5 molecules.

Molecule	Lipinski	Ghose	Veber	Egan	Muegge	Bioavailability Score	PAINS
MAD1	Yes	Yes	Yes	Yes	Yes	0.55	1 alert: ene_six_het_A
MAD2	Yes	No; 1 violation: WLOGP < −0.4	Yes	Yes	Yes	0.56	1 alert: ene_six_het_A
MAD3	Yes	Yes	Yes	Yes	Yes	0.55	1 alert: ene_six_het_A
MAD4	Yes	Yes	Yes	Yes	Yes	0.55	1 alert: ene_six_het_A
MAD5	Yes	No; 1 violation: WLOGP < −0.4	Yes	Yes	Yes	0.56	1 alert: ene_six_het_A

**Table 6:** Predicted toxicological profile of five acids derived from Meldrum’s (MAD1–MAD5), obtained through the ProTox 3.0 online platform.

Classification	Target	Prediction (Compounds)				
		MAD1	MAD2	MAD3	MAD4	MAD5
Organ toxicity	Hepatotoxicity	Inactive	Active	Inactive	Inactive	Active
	Neurotoxicity	Inactive	Inactive	Inactive	Inactive	Inactive
	Nephrotoxicity	Active	Active	Active	Active	Active
	Respiratory toxicity	Active	Active	Active	Active	Active
	Cardiotoxicity	Inactive	Inactive	Inactive	Inactive	Inactive
Toxicity end points	Carcinogenicity	Inactive	Inactive	Inactive	Inactive	Inactive
	Immunotoxicity	Inactive	Inactive	Inactive	Inactive	Inactive
	Mutagenicity	Inactive	Inactive	Inactive	Inactive	Inactive
	Cytotoxicity	Inactive	Inactive	Inactive	Inactive	Inactive
	BBB-barrier	Active	Active	Active	Active	Active
	Ecotoxicity	Inactive	Active	Active	Active	Inactive
	Clinical toxicity	Active	Active	Active	Active	Active
	Nutritional toxicity	Active	Active	Active	Active	Active
Nuclear receptor signalling pathways	Aryl hydrocarbon Receptor (AhR)	Inactive	Inactive	Inactive	Inactive	Inactive
	Androgen Receptor (AR)	Inactive	Inactive	Inactive	Inactive	Inactive
	Androgen Receptor Ligand Binding Domain (AR-LBD)	Inactive	Inactive	Inactive	Inactive	Inactive
	Aromatase	Inactive	Inactive	Inactive	Inactive	Inactive
	Estrogen Receptor Alpha (ER)	Inactive	Inactive	Inactive	Inactive	Inactive
	Estrogen Receptor Ligand Binding Domain (ER-LBD)	Inactive	Inactive	Inactive	Inactive	Inactive
	Peroxisome Proliferator Activated Receptor Gamma (PPAR-Gamma)	Inactive	Inactive	Inactive	Inactive	Inactive
Stress response pathways	Nuclear factor (erythroid-derived 2)-like 2/antioxidant responsive element (nrf2/ARE)	Inactive	Inactive	Inactive	Inactive	Inactive
	Heat shock factor response element (HSE)	Inactive	Inactive	Inactive	Inactive	Inactive
	Mitochondrial Membrane Potential (MMP)	Inactive	Inactive	Inactive	Inactive	Inactive
	Phosphoprotein (Tumor Supressor) p53	Inactive	Inactive	Inactive	Inactive	Inactive
	ATPase family AAA domain-containing protein 5 (ATAD5)	Inactive	Inactive	Inactive	Inactive	Inactive
Molecular Initiating Events	Thyroid hormone receptor alpha (THRα)	Inactive	Inactive	Inactive	Inactive	Inactive
	Thyroid hormone receptor beta (THRβ)	Inactive	Inactive	Inactive	Inactive	Inactive
	Transtyretrin (TTR)	Inactive	Inactive	Inactive	Inactive	Inactive
	Ryanodine receptor (RYR)	Inactive	Inactive	Inactive	Inactive	Inactive
	GABA receptor (GABAR)	Inactive	Inactive	Inactive	Inactive	Inactive
	Glutamate N-methyl-D-aspartate receptor (NMDAR)	Inactive	Inactive	Inactive	Inactive	Inactive
	alpha-amino-3-hydroxy-5-methyl-4-isoxazolepropionate receptor (AMPAR)	Inactive	Inactive	Inactive	Inactive	Inactive
	Kainate receptor (KAR)	Inactive	Inactive	Inactive	Inactive	Inactive
	Achetylcholinesterase (AChE)	Inactive	Inactive	Inactive	Inactive	Inactive
	Constitutive androstane receptor (CAR)	Inactive	Inactive	Inactive	Inactive	Inactive
	Pregnane X receptor (PXR)	Inactive	Inactive	Inactive	Inactive	Inactive
	NADH-quinone oxidoreductase (NADHOX)	Inactive	Inactive	Inactive	Inactive	Inactive
	Voltage gated sodium channel (VGSC)	Inactive	Inactive	Inactive	Inactive	Inactive
	Na+/I- symporter (NIS)	Inactive	Inactive	Inactive	Inactive	Inactive
Metabolism	Cytochrome CYP1A2	Inactive	Inactive	Inactive	Inactive	Inactive
	Cytochrome CYP2C19	Inactive	Inactive	Inactive	Inactive	Inactive
	Cytochrome CYP2C9	Inactive	Inactive	Active	Active	Inactive
	Cytochrome CYP2D6	Inactive	Inactive	Inactive	Inactive	Inactive
	Cytochrome CYP3A4	Inactive	Inactive	Inactive	Inactive	Inactive
	Cytochrome CYP2E1	Inactive	Inactive	Inactive	Inactive	Inactive

**Table 7:** Meldrum’s Acid Derivatives.

Systematic Name	Molecular Structure	Acronym
N-{6-[(2,2-Dimethyl-4,6-dioxo-[1,3]dioxane-5-ylidenemethyl)-amino]-pyridin-2-yl}-acetamide)	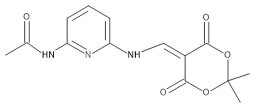	MAD1
2,2-dimethyl-5-[(1-methyltetrazol-5-ylamino)methylene]-1,3-dioxane-4,6-dione)	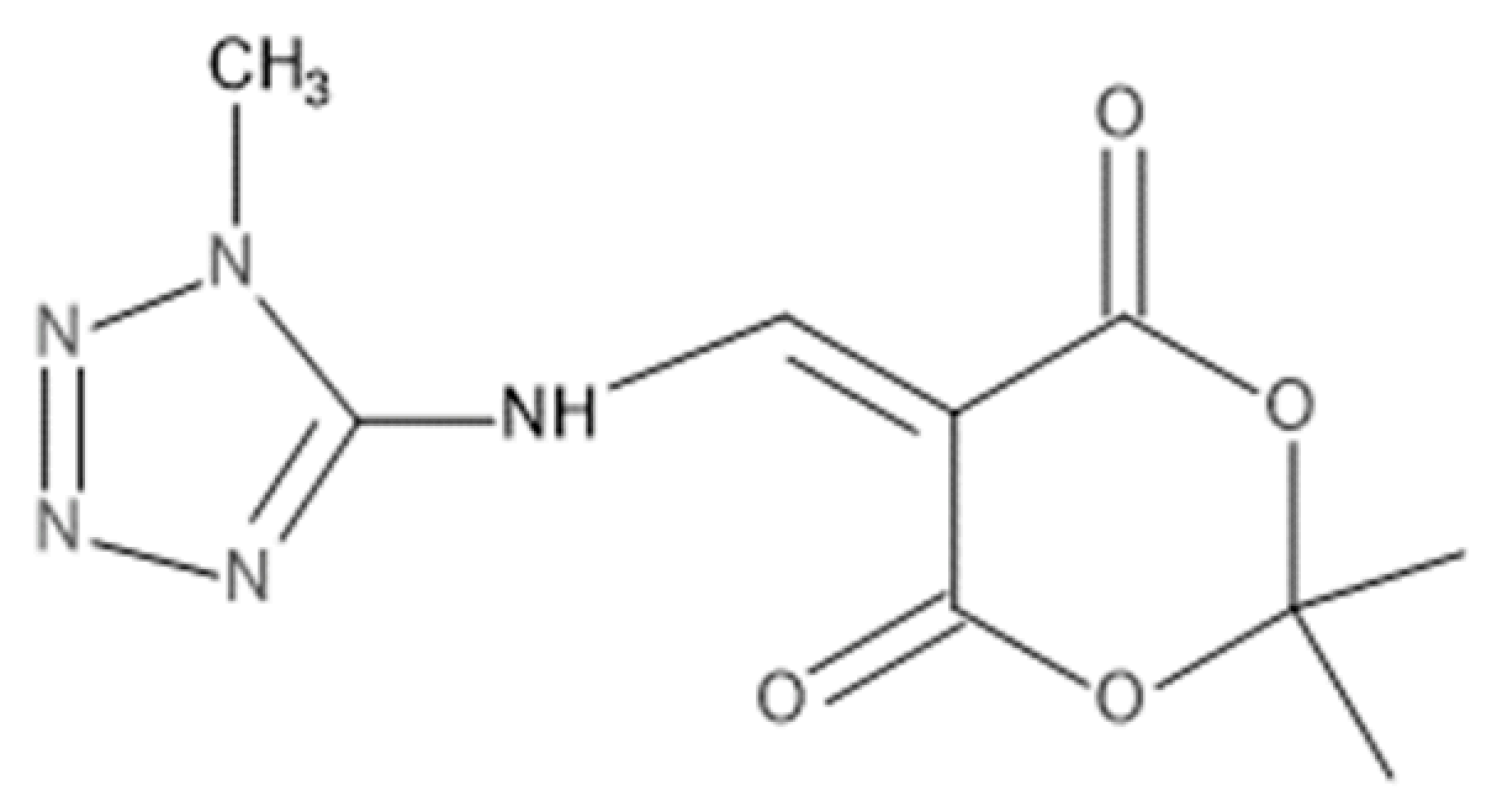	MAD2
5-[(4,6-dimethyl-pyridin-2-ylamino)-methylene]-2,2-dimethyl-[1,3]dioxan-4,6-dione	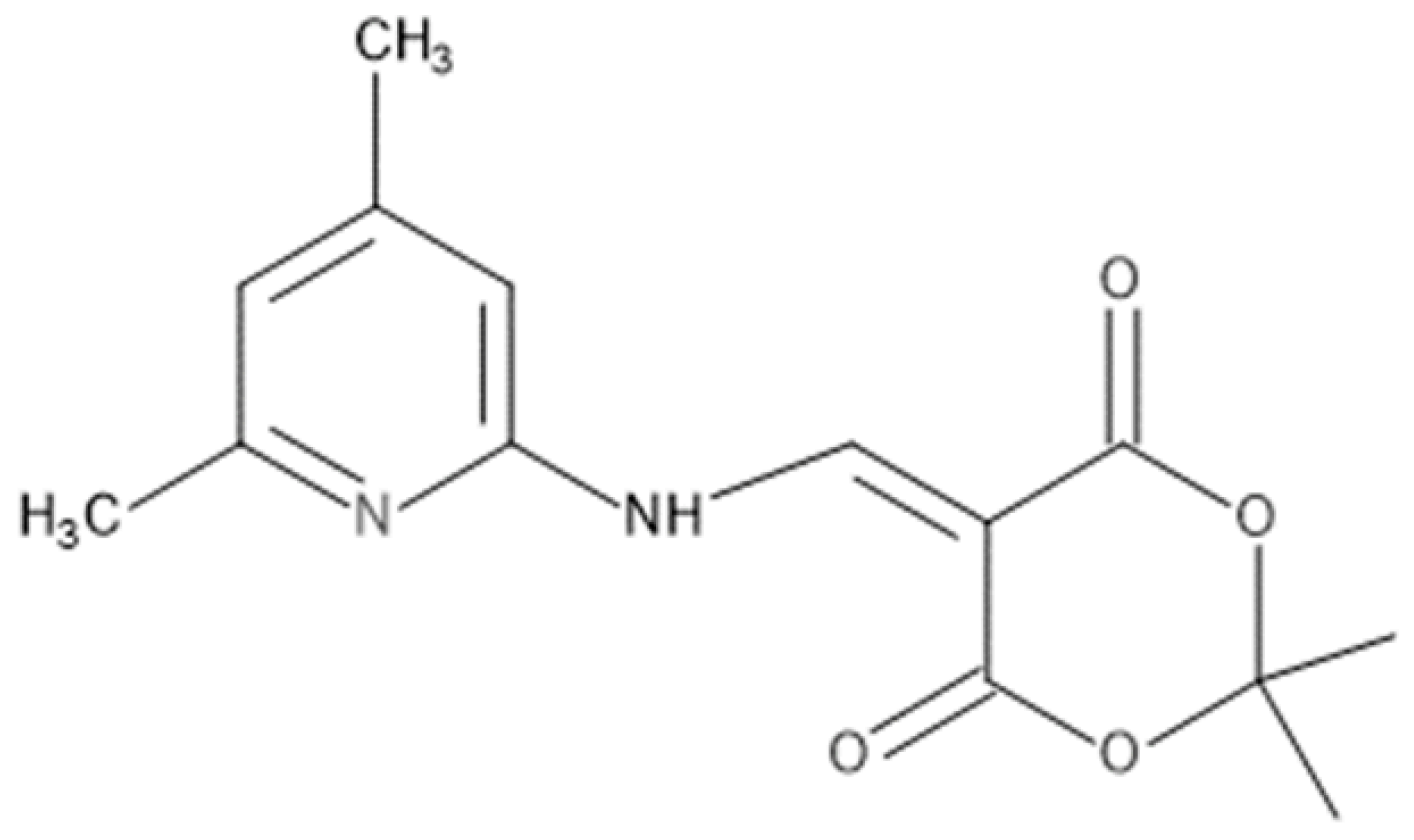	MAD3
2,2-dimethyl-5-[(6-methyl-pyridin-2-ylamino)-methylene]-1,3-dioxan-4,6-dione	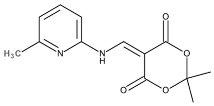	MAD4
2,2-dimethyl-5-[(tetrazol-5-ylamino)methylene]-1,3-dioxane-4,6-dione	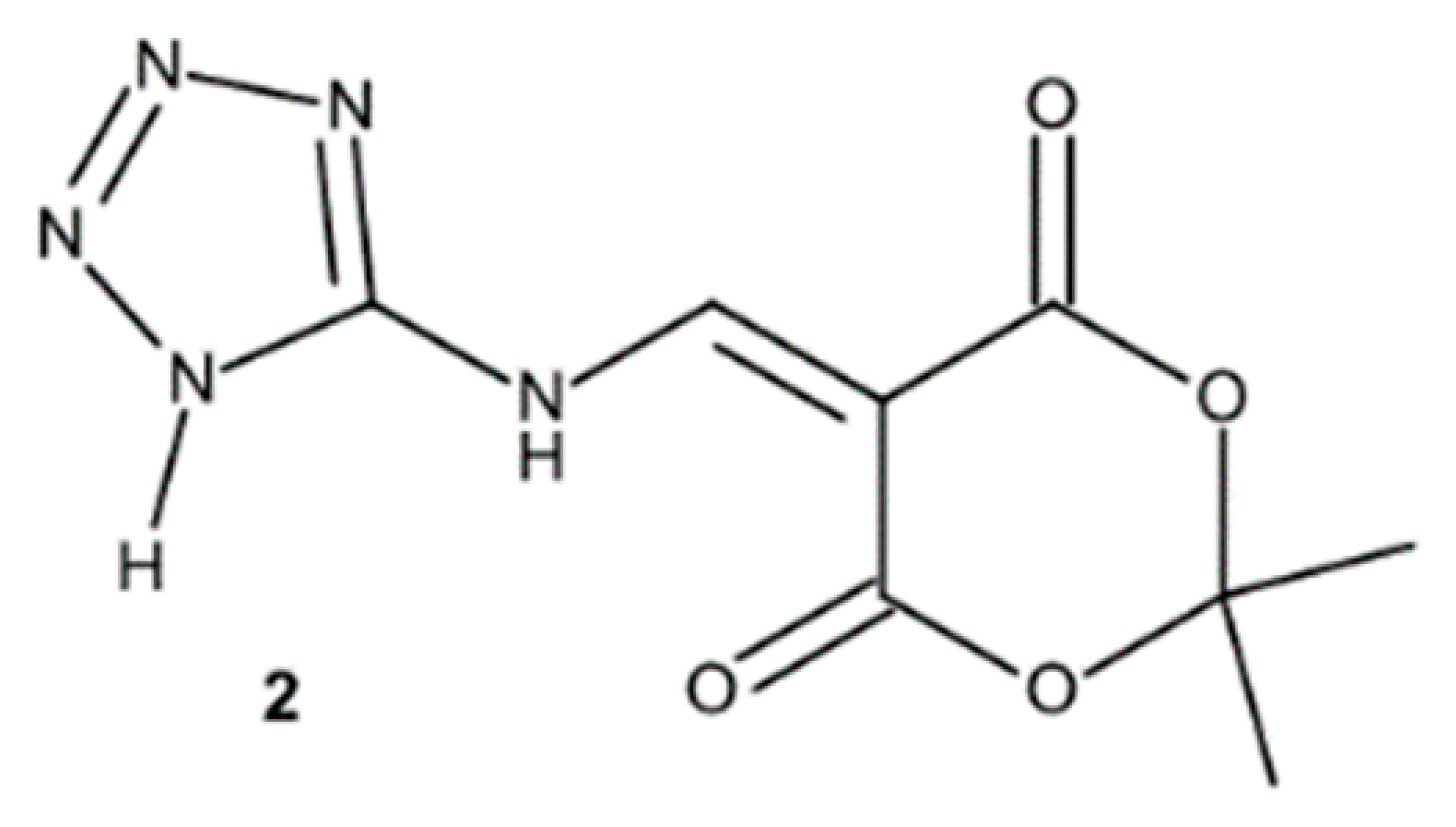	MAD5

MAD—Meldrum’s Acid Derivatives.

**Table 8:** SMILES codes used in ADMET prediction.

Code	SMILES
MAD1	O=C1OC(C)(C)OC(/C1=C\NC2=NC(NC(C)=O)=CC=C2)=O
MAD2	O=C1OC(C)(C)OC(/C1=C\NC2=NN=NN2[CH3])=O
MAD3	CC1=CC(N/C=C2C(OC(C)(C)OC\2=O)=O)=NC(C)=C1
MAD4	CC1=CC=CC(N/C=C2C(OC(C)(C)OC\2=O)=O)=N1
MAD5	O=C1OC(C)(C)OC(/C1=C\NC2=NN=NN2[H])=O

## Data Availability

The data that support the findings of this study are available from the corresponding author upon reasonable request.

## Abbreviations

The following abbreviations are used in this manuscript:

ADMET	Absorption, Distribution, Metabolism, Excretion and Toxicity
ANOVA	Analysis of Variance
ATP	Adenosine Triphosphate
BBB	Blood–Brain Barrier
BHI	Brain Heart Infusion
CCCP	Carbonyl Cyanide m-Chlorophenylhydrazone
CLSI	Clinical and Laboratory Standards Institute
CYP	Cytochrome P450
DMSO	Dimethyl Sulfoxide
DNA	Deoxyribonucleic Acid
FLEX	Molecular Flexibility
GI	Gastrointestinal
HA	Heavy Atoms
INSATU	Unsaturation
INSOLU	Insolubility
LIPO	Lipophilicity
LPS	Lipopolysaccharide
MAD	Meldrum’s Acid Derivative
MDR	Multidrug-Resistant
MIC	Minimum Inhibitory Concentration
MMP	Mitochondrial Membrane Potential
MRSA	Methicillin-Resistant *Staphylococcus aureus*
NIS	Sodium/Iodide Symporter
NMDAR	N-Methyl-D-Aspartate Receptor
PAβN	Phenylalanine-Arginine β-Naphthylamide
PAINS	Pan-Assay Interference Compounds
PBS	Phosphate-Buffered Saline
P-gp	P-Glycoprotein
PPAR	Peroxisome Proliferator-Activated Receptor
PXR	Pregnane X Receptor
RLU	Relative Luminescence Units
RYR	Ryanodine Receptor
SMILES	Simplified Molecular Input Line Entry System
SYTOX Green	SYTOX Green nucleic acid stain
TPSA	Topological Polar Surface Area
UPEC	Uropathogenic *Escherichia coli*
VGSC	Voltage-Gated Sodium Channel
WHO	World Health Organization
